# Uterine Microbiota Composition in Dairy Cows with Different Vaginal Discharge Scores: Suggesting Caviibacter as a Potential Pathogen in Mild Purulent Metritis

**DOI:** 10.3390/microorganisms13081728

**Published:** 2025-07-24

**Authors:** Xiaolei He, Jiajia Wang, Lin Jiang, Xinyu Wang, Yuxing Wang, Yang Liu, Yanping Cheng, Fei Xu, Xiubo Li

**Affiliations:** 1 Institute of Feed Research, Chinese Academy of Agricultural Sciences, Beijing 100081, China; hxlei200005@163.com (X.H.); jiajiawang2025@outlook.com (J.W.); jianglin0046@163.com (L.J.); mihoutao1996@163.com (X.W.); xywang0411@163.com (Y.W.); ly1250921848@163.com (Y.L.); 2 Beijing Lvhe Cattle Farming Co., Ltd., Beijing 100163, China; cyp041109@163.com

**Keywords:** postpartum metritis, uterine microbiota, vaginal discharge

## Abstract

The uterine microbiota plays a crucial role in maintaining postpartum reproductive health in dairy cows, and its dysregulation is closely associated with uterine diseases. Vaginal discharge characteristics serve as important clinical indicators for assessing uterine status and guiding clinical decision-making. This study employed 16S rRNA gene sequencing to analyze uterine microbial diversity in cows with different discharge types. Results revealed significant microbial shifts associated with discharge severity. Notably, *Caviibacter* was highly enriched (up to 60.25%) in cows with mildly purulent discharge (<50%), suggesting its potential role in early-stage endometritis. In contrast, *Fusobacterium* and *Helcococcus* dominated when purulent discharge exceeded 50%, while *Bacteroides*, *Porphyromonas*, and *Peptostreptococcus* prevailed in cows with malodorous or discolored secretions, indicating severe inflammation. This study extends previous findings by uncovering stage-specific microbial transitions and proposing *Caviibacter* as a potential early biomarker of endometritis. These insights support early diagnosis and targeted interventions, contributing to improved reproductive management and sustainable dairy farming.

## 1. Introduction

Postpartum metritis in dairy cows is a common and significant disease in the dairy farming industry, severely impacting reproductive performance and milk production, leading to substantial economic losses for farmers. Clinically, it is characterized by the presence of purulent or mucopurulent vaginal discharge in dairy cows after parturition, with severe cases exhibiting slightly reddish or brownish discharge accompanied by foul-smelling watery fluid [1]. During the dairy farming process, the Metricheck tool is used to collect vaginal discharge from postpartum cows, and the discharge is scored, which serves as an important method for the clinical diagnosis of bovine metritis [2,3]. The characteristics and severity of vaginal discharge are closely associated with the postpartum interval. Previous studies have reported that abnormal discharge typically peaks between 14 and 21 days after calving, which represents a critical window for detecting uterine involution abnormalities and infection-related conditions such as metritis. Therefore, in the clinical diagnosis of uterine health status, it is essential for veterinarians to consider both the number of days postpartum and the vaginal discharge score as combined factors for accurate assessment. Increasing research has shown that the uterus also harbors a microbiome, and specific microbial communities may be linked to the oral cavity and gastrointestinal mucosa, as well as potentially being influenced by the vaginal microbiome [4]. The balance of the uterine microbiota is crucial for uterine health, with a healthy microbiome maintained by beneficial bacteria and potential pathogens, which together help regulate the host’s immune system. However, when the microbial community becomes imbalanced, uterine diseases are more likely to occur [5]. In fact, this phenomenon is also observed in dairy cows, with evidence showing that both virgin and pregnant cows have a resident uterine microbiome until the periparturient period, when the uterine microbial equilibrium is disrupted. The establishment of the postpartum uterine microbiome in cows may be related to retrograde colonization from the rectum and vagina [6,7].

In recent years, advancements in sequencing technologies have provided a more comprehensive and in-depth perspective for analyzing the uterine microecological environment in cows with metritis [8]. Sequencing analyses have shown that *Firmicutes*, *Bacteroidetes*, and *Proteobacteria* are the dominant phyla in the uterine microbiome of dairy cows. Notably, cows with metritis exhibit significant differences in the composition of the uterine bacterial community compared to healthy postpartum cows [9,10]. Furthermore, further investigation has revealed that *Bacteroides*, *Porphyromonas*, and *Fusobacterium* are pathogenic microorganisms closely associated with metritis [8]. To our knowledge, while existing studies have indicated that the imbalance of the uterine microbiota is closely related to the occurrence of metritis, most studies analyze cows with metritis as a whole, lacking an in-depth exploration of the differences in uterine microbiota across varying vaginal mucus scores. Therefore, this study analyzes the uterine microenvironment of dairy cows with different vaginal discharge states to explore the relationship between this microenvironment and bovine metritis, with the goal of providing a basis for clinical diagnosis and treatment strategies. This study focuses on postpartum cows with different discharge states and uses 16S rRNA gene sequencing technology to explore the structural and compositional differences in microbial communities.

## 2. Materials and Methods

### 2.1. Feeding Management

This study was conducted in August 2024 at a dairy farm in Tongzhou, Beijing. All experiments were conducted in strict accordance with relevant laws, regulations, and guidelines and were approved by the Laboratory Animal Ethics Committee of the Institute of Feed Research of the Chinese Academy of Agricultural Sciences and its inspections. The dairy farm had 2024 Holstein dairy cows, of which 946 were adult females, and the average daily milk production of the herd was 32 tons. Before and after the experiments, the cows were routinely cared for in a 120 m long, 76 m wide, roofed enclosure. The pens were equipped with fans and automatic sprinklers. Before and after the trial, the cows were fed a multi-mineral and vitamin concentrate and a mixture of roughage such as alfalfa, corn flakes, and brewer’s grains to meet their nutritional requirements, while maintaining an uninterrupted supply of water.

### 2.2. Test Grouping

**Figure 1:**
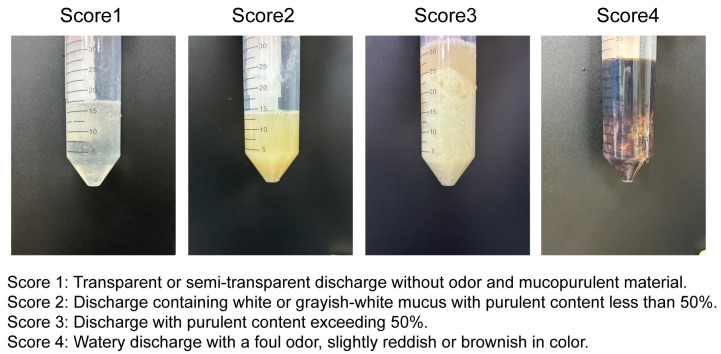
The figure shows the vaginal discharge of dairy cows scored from 1 to 4.

The inclusion criteria for the trial cows were Holstein cows diagnosed by a licensed veterinarian with no clinical diseases other than metritis and no recent history of medication that could interfere with the study. Cows were excluded if they developed additional disease symptoms during the trial that required antimicrobial or anti-inflammatory treatment. All cows were evaluated at a standardized time point—specifically 15 days postpartum (DPP)—to ensure consistency in discharge scoring and to eliminate potential confounding effects of postpartum time variation. At this time point, vaginal discharge was assessed using the Metricheck device (Simcro, Hamilton, New Zealand), a widely used and effective tool for evaluating postpartum reproductive health in dairy cows. The device consists of a stainless steel rod with a rubber hemisphere at the end and enables the collection and visual assessment of vaginal discharge. Compared to manual examination, it offers a more standardized and objective evaluation of discharge characteristics, including color, consistency, and odor. Based on the specific management conditions of the local dairy farm, the vaginal discharge scoring system was defined as follows: transparent or semi-transparent discharge without odor and without mucopurulent material was scored as 1; discharge containing white or grayish-white mucus with purulent content less than 50% was scored as 2; discharge with purulent content exceeding 50% was scored as 3; and watery discharge with a foul odor, slightly reddish or brownish in color, was scored as 4. This scoring system is illustrated in Figure 1. Based on the scoring system (from score 1 to score 4), four cows with similar age, body weight, and parity were randomly selected from each score group to form Group 1, Group 2, Group 3, and Group 4, respectively. Following strict selection criteria, a total of 16 Holstein cows were included in the subsequent trial.

### 2.3. Sample Collection

Before sampling, the perineal area of the cows was thoroughly washed with water and sterilized under the guidance of the clinical veterinarian of the farm. During the sampling process, a technician used a sterile uterine swab to collect uterine secretions from each group of test cows and then transferred the collected samples to a sterile centrifuge tube and numbered and recorded them. The operation was standardized throughout the operation to avoid sample contamination. After the samples were collected, they were immediately placed on ice for transportation and quickly transferred to the laboratory for storage in a −80 °C refrigerator for further processing. Prior to 16S rRNA sequencing, preliminary microbial isolation and culture combined with microscopic examination were performed in the laboratory to rule out protozoal and fungal infections in the reproductive tract, ensuring the reliability and validity of the experimental results.

### 2.4. DNA Extraction and Sequencing

Microbial community genomic DNA was extracted using the Qiagen QIAamp DNA Mini Kit (QIAGEN, Hilden, Germany) and the quality, concentration, and purity of the DNA were assessed using 1% agarose gel electrophoresis and a NanoDrop 2000 spectrophotometer (Thermo Fisher Scientific, Waltham, MA, USA). The 16S rRNA gene V3-V4 region was amplified using primers 338F and 806R with barcode sequences. The PCR reaction mixture included buffer, dNTPs, primers, DNA polymerase, and template DNA. After amplification, the PCR products were purified using agarose gel electrophoresis, and the concentration was quantified using a Qubit 4.0 fluorometer (Thermo Fisher Scientific, Waltham, MA, USA). Subsequently, libraries were constructed using the NEXTFLEX Rapid DNA-Seq Kit (PerkinElmer, Waltham, MA, USA), and sequencing was performed on the Illumina Nextseq2000 platform (Illumina, San Diego, CA, USA).

### 2.5. Bioinformatics Analysis

For the paired-end (PE) reads generated by Illumina sequencing, the sequences were first subjected to assembly, quality control, and filtering. ASVs (Amplicon Sequence Variants) were annotated for taxonomic classification, and diversity indices were calculated to verify whether the sequencing depth was sufficient. Using taxonomic information, the structural characteristics of the microbial community were analyzed, and taxonomic composition and differences between groups were examined. The results were presented in a visual format.

Rank–abundance curves were generated using Python (v2.7) at the genus level to assess species richness and community evenness. Rarefaction curves based on the Sobs index were produced in mothur (v1.30) at the genus level to evaluate sequencing depth sufficiency. Alpha diversity metrics, including Chao1 and Shannon indices, were calculated at the ASV level using mothur (v1.30), and differences among groups (Group 1 to Group 4) were assessed with Kruskal–Wallis tests followed by post hoc Tukey–Kramer comparisons and FDR correction in R (v3.3.1). Beta diversity was explored via Principal Coordinate Analysis (PCoA) based on Bray–Curtis distances calculated using the vegan package in R (v3.3.1), with group differences tested by ANOSIM (999 permutations). Microbial community composition was visualized through Venn diagrams and stacked bar plots generated in Python (v2.7). Taxa ranked below the top 30 in abundance or unclassified taxa were grouped as “Others.” Multi-group differential abundance analysis at the genus level was conducted in R using Kruskal–Wallis tests with post hoc Tukey–Kramer comparisons and FDR correction. Finally, LEfSe analysis was performed in R from phylum to genus levels with an LDA score threshold of 4.0 to identify key taxa discriminating between groups.

## 3. Results

### 3.1. Differences in Uterine Microbial Diversity and Richness Across Different Vaginal Discharge Scores

**Figure 2:**
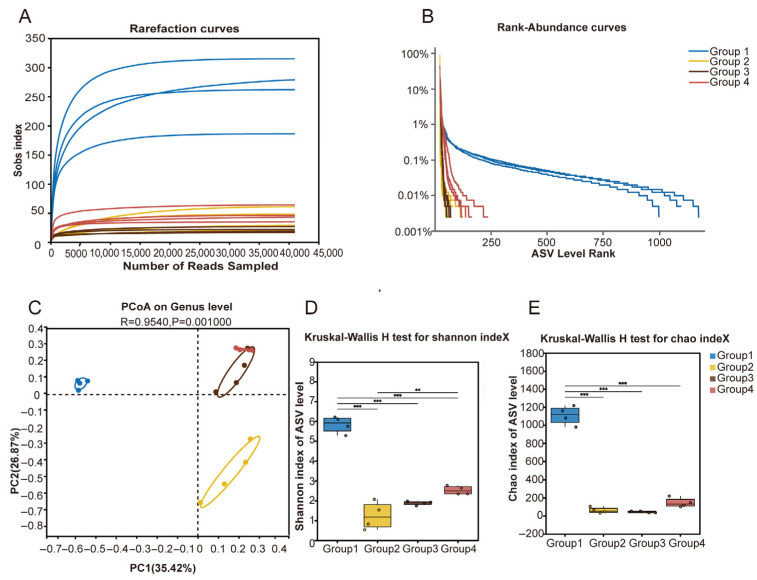
(**A**) The dilution curve is used to assess whether the sequencing data are sufficient. When the curve flattens at the end, it indicates that the sequencing data is reasonable. (**B**) Rank–abundance curve showing taxonomic richness and evenness. The width of the curve along the x-axis reflects the richness of observed taxa—the broader the curve, the higher the richness. (**C**,**D**) Kruskal–Wallis H tests based on Chao1 and Shannon indices, respectively, calculated using mothur (v1.30), display significant differences between groups. Significant differences between two groups are marked (** 0.001 < *p* ≤ 0.01, *** *p* ≤ 0.001). (**E**) Principal Coordinate Analysis (PCoA) plot based on genus-level data, generated using the vegan package in R (v3.3.1), showing differences in microbial community structures between sample groups. Different colors or shapes represent different groups; closer points indicate more similar communities.

To analyze the uterine microbiota in cows with different uterine discharge scores, we performed 16S rRNA sequencing and diversity data analysis on 16 samples. A total of 1,299,246 optimized sequences were obtained, with 536,777,873 bases and an average sequence length of 413 bp. A certain number of sequences were randomly selected from the samples, and the alpha diversity indices for the corresponding samples were calculated. We plotted a dilution curve with the number of sequences extracted on the x-axis and the alpha diversity index on the y-axis. The results showed that the end of the dilution curve for all samples tended to flatten, indicating that the sequencing data was reasonable and the sequencing depth was sufficient to cover the overall bacterial diversity (Figure 2A).

On the horizontal axis, the width of the rank–abundance curve reflects the abundance of ASVs. The broader the range of the curve on the x-axis, the higher the ASV abundance. From the figure, it can be seen that the curve for Group 1 descends more gradually, indicating a more even distribution of ASV relative abundance in Group 1. In contrast, the curves for Group 2, Group 3, and Group 4 descend more steeply, suggesting the dominance of a few ASVs and lower community evenness in these groups (Figure 2B). Furthermore, the box plots of alpha diversity indices—Chao1 (richness) and Shannon (diversity)—demonstrated that both microbial richness and diversity were significantly reduced in groups with a vaginal discharge score ≥ 2 compared to Group 1 (Figure 2C,D).

Principal Coordinate Analysis (PCoA), based on Bray–Curtis distances of genus-level abundance data, was used to assess differences in the uterine microbiota among cows with different vaginal discharge scores. PCoA is a dimensionality reduction technique that transforms complex distance matrices into visualizable axes, allowing clear comparison of microbial community structures between samples. The results revealed that one sample from Group 3 clustered the groups (*p* = 0.001), indicating that the microbial compositions differed significantly across discharge score categories (Figure 2E).

### 3.2. Differences in Uterine Microbial Composition Across Different Vaginal Discharge Scores

**Figure 3:**
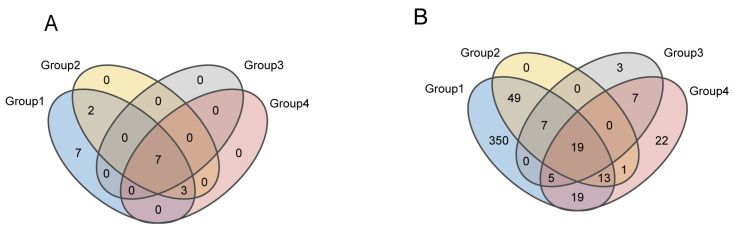
(**A**,**B**) Venn diagrams of the uterine microbiota at the phylum and genus levels in cows from different groups. Different colors represent different groups. Overlapping regions indicate taxa (at the phylum or genus level) that are shared among multiple groups, while non-overlapping areas represent taxa unique to a specific group. Numbers within each area correspond to the count of taxa.

**Figure 4:**
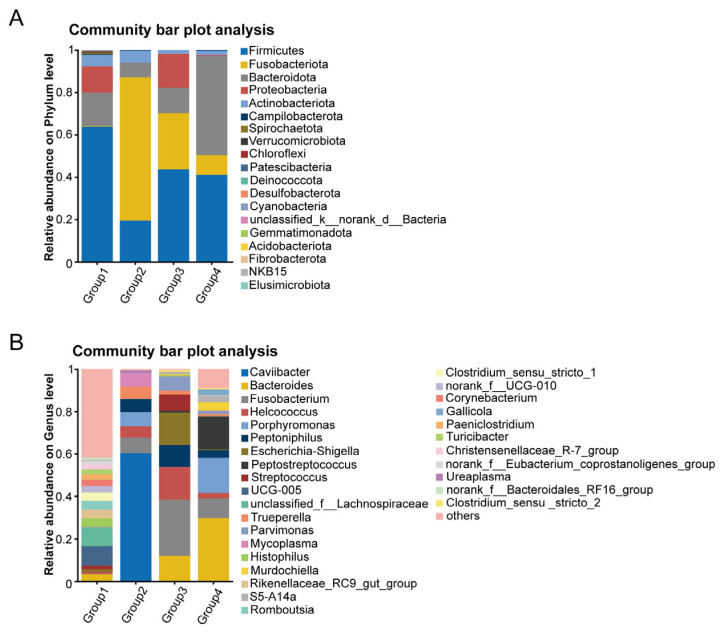
(**A**,**B**) Community bar plots of the uterine microbiota at the phylum (**A**) and genus (**B**) levels across groups. These bar plots show the average microbial composition of the top 30 taxa ranked by relative abundance in each group. Taxa with low abundance were combined under “Others.” The plots illustrate changes in dominant taxa among the groups. Each color represents a different taxon, and the length of each colored bar indicates its average relative abundance in the corresponding group.

To further investigate the differences in the uterine microbiota among different score groups, we performed a community composition analysis. Using a Venn diagram, we calculated the number of shared and unique genera across the groups. We found that all groups shared 19 bacterial genera. Group 1 had 350 unique genera, Group 2 had 5 unique genera, Group 3 had 3 unique genera, and Group 4 had 22 unique genera (Figure 3A,B). The community bar plot displayed the relative abundance of microorganisms at the phylum and genus levels, with a focus on the top 10 phyla and genera. The results confirmed previous studies, showing that at the phylum level, the dominant phyla in Group 1 were *Firmicutes* (63.85%), *Bacteroidota* (15.78%), *Proteobacteria* (12.35%), and *Actinobacteriota* (5.50%). In Group 2, the dominant phyla were *Fusobacteriota* (67.60%), *Firmicutes* (19.47%), *Bacteroidota* (6.84%), and *Actinobacteriota* (5.73%). In Group 3, the dominant phyla were *Firmicutes* (43.68%), *Fusobacteriota* (26.56%), *Proteobacteria* (15.95%), *Bacteroidota* (11.99%), and *Actinobacteriota* (1.81%). In Group 4, the dominant phyla were *Bacteroidota* (46.95%), *Firmicutes* (41.03%), *Fusobacteriota* (9.36%), and *Actinobacteriota* (2.02%) (Figure 4A).

At the genus level, significant differences were observed in the uterine microbial communities across the four groups. In Group 1, the top five most abundant genera were *UCG-005* (9.23%), *unclassified_f_Lachnosoiraceae* (8.81%), *Rikenellaceae_RC9_gut_group* (4.21%), *Romboutsia* (4.10%), *Histophilus* (3.91%), and *Clostridium_sensu_stricto_1* (3.91%). Notably, in Group 2, the relative abundance of *Caviibacter* reached 60.25%, with other genera exhibiting relative abundances greater than 5% including *Fusobacterium* (7.35%), *Porphyromonas* (6.74%), *Mycoplasma* (6.21%), *Peptoniphilus* (6.03%), *Trueperella* (5.73%), and *Helcococcus* (5.35%). In Group 3, the top five most abundant genera were *Fusobacterium* (26.41%), *Helcococcus* (15.36%), *Escherichia-Shigella* (15.29%), *Bacteroides* (11.95%), and *Peptoniphilus* (10.39%). In Group 4, the top five most abundant genera were *Bacteroides* (29.69%), *Peptostreptococcus* (15.39%), *Fusobacterium* (9.36%), and *Murdochiella* (3.85%) (Figure 4B).

### 3.3. Differential Microbial Communities in the Uterus of Cows with Different Vaginal Discharge Scores

**Figure 5:**
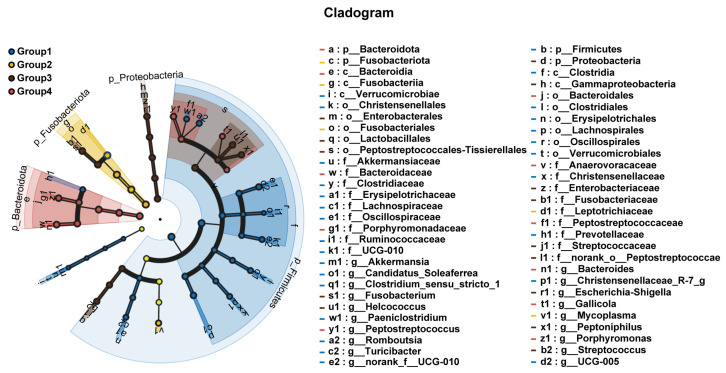
Cladogram illustrating differences in microbial composition from the phylum to genus levels across groups. This cladogram visually displays microbial taxa differences from the phylum to genus levels between groups. Different colored nodes represent microbial taxa that are significantly enriched in the corresponding groups and have a significant impact on inter-group differences. Light yellow nodes indicate microbial taxa with no significant differences between groups or that have no significant impact on inter-group differences. (The letter before the name represents the taxonomic unit: g = genus, f = family, o = order.)

To further explore the differential microbial communities in the uterine microbiota under different vaginal discharge scores, we performed Linear Discriminant Analysis Effect Size (LEfSe) analysis. This analysis identified several taxa with significantly different abundances between groups, based on non-parametric statistical testing and LDA score ranking. The results indicated that the bacterial communities with significantly different abundances across the four groups included 8 phyla, 12 classes, 20 orders, 28 families, and 31 genera (*p* < 0.05, LDA score > 4.0). The Linear Discriminant Analysis (LDA) score reflects the effect size of each differentially abundant taxon, with higher scores indicating greater contribution to the differences among groups. From Figure 5, we observed that at the phylum level, *Fusobacteriota*, *Bacteroidota*, *Firmicutes*, and *Proteobacteria* showed significant differences across groups. Specifically, *Firmicutes* was enriched in the uterine microbiota of cows with a vaginal discharge score of 1, *Proteobacteria* was enriched in cows with a score of 2, *Fusobacteriota* was enriched in cows with a score of 3, and *Bacteroidota* was enriched in cows with a score of 4.

**Figure 6:**
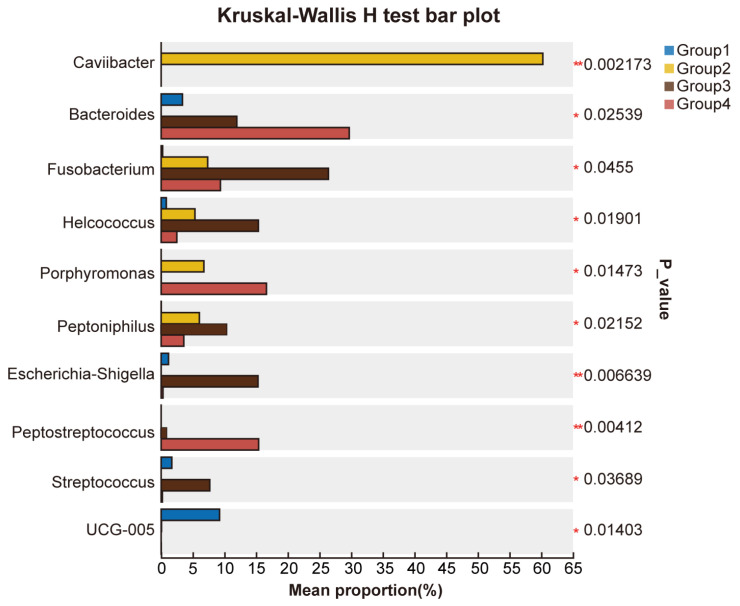
Comparison of the average relative abundance of microbial genera across different groups. This bar plot displays the differences in the average relative abundance of the same genus among multiple groups. Different colors represent different groups. The *p*-values on the far right indicate the statistical significance, with asterisks marking significant differences: * 0.01 < *p* ≤ 0.05, ** 0.001 < *p* ≤ 0.01.

**Figure 7:**
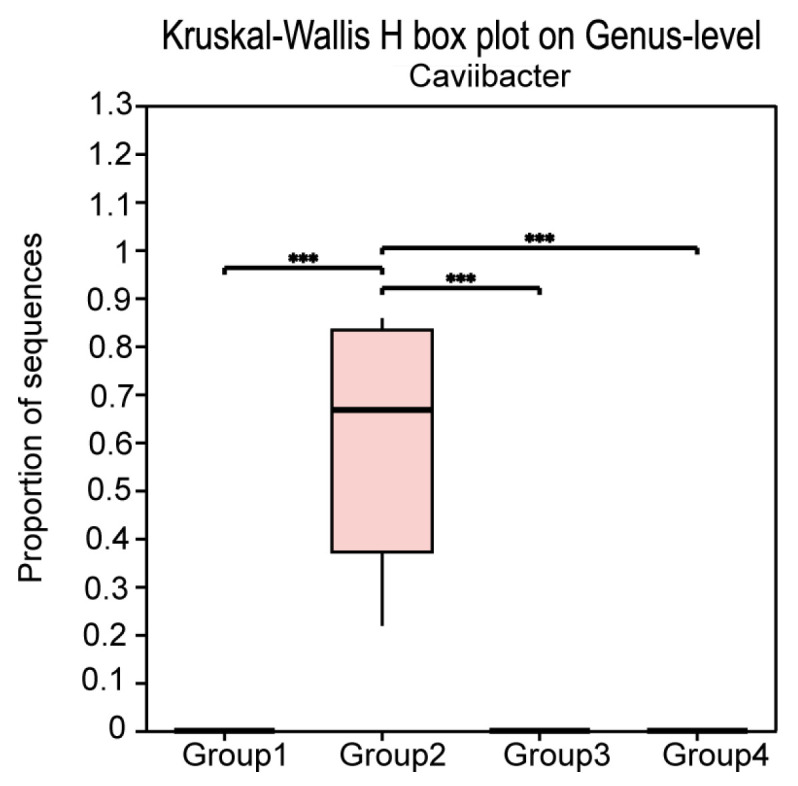
Boxplot showing the relative abundance of Caviibacter at the genus level, analyzed using the Kruskal–Wallis H test implemented in R (v3.3.1). Asterisks indicate significant differences *** *p* ≤ 0.001.

Furthermore, at the genus level, we used the Kruskal–Wallis rank-sum test to select the top ten bacterial genera in terms of relative abundance (*p*-value < 0.05) and compared their relative abundance across groups. The results showed that in Group 1, the relative abundance of *UCG-005*—a genus-level taxon within the family *Ruminococcaceae*, commonly identified in the ruminant gut and reproductive tract microbiota—was significantly higher than in the other three groups. In Group 2, the relative abundance of *Caviibacter* was significantly higher than in the other groups. In Group 3, the relative abundance of *Fusobacterium*, *Helcococcus*, *Peptoniphilus*, *Escherichia-Shigella*, and *Streptococcus* was significantly increased. In Group 4, the relative abundance of *Bacteroides*, *Porphyromonas*, and *Peptostreptococcus* was significantly increased (Figure 6). Notably, *Caviibacter* was exclusively enriched in cows with mildly purulent discharges (<50%), whereas it was undetectable in both healthy cows and those with more severe purulent discharges and pronounced clinical symptoms (Figure 7).

## 4. Discussion

Exploring the microbial community characteristics of diseased dairy cows can enhance our understanding of the pathogenesis of metritis. Combining these findings with clinical diagnostic measures has significant implications for the prevention and treatment of this condition. In this study, we used the status of vaginal discharge as a clinical scoring criterion to investigate the microbial community structure and characteristics of the uterine microbiota in dairy cows with different discharge scores, aiming to provide additional data to support the clinical management of bovine metritis. Through analysis of 16S rRNA sequencing results, we observed that compared to postpartum healthy cows, cows with purulent and foul-smelling watery vaginal discharge had significantly lower uterine microbial diversity and richness. This could be due to the inflammatory environment suppressing microbial diversity, with the proliferation of pathogenic bacteria leading to a reduction in healthy microbiota [7,11,12,13].

Further analysis revealed that the uterine microbiota of postpartum dairy cows with different vaginal discharge states exhibited both overlapping and distinct differences. At the phylum level, *Firmicutes*, *Fusobacteriota*, *Bacteroidota*, and *Proteobacteria* were found to be the predominant phyla in the uterine microbiota of cows in all groups, which is consistent with previous studies [14]. Other studies have reported a higher abundance of *Fusobacteriota* and *Bacteroidota* and lower abundance of *Firmicutes* and *Proteobacteria* in cows with metritis compared to healthy cows [15]. Our findings add new insights, with cows exhibiting a vaginal discharge score of 4 and foul-smelling watery discharge showing this trend. However, in cows with vaginal discharge scores of 2 and 3, the relative abundance of *Bacteroidota* was lower than in healthy cows (score 1). Furthermore, cows with a discharge score of 3 had a higher relative abundance of *Proteobacteria* compared to healthy cows.

At the genus level, it is noteworthy that in cows with less than 50% purulent discharge, *Caviibacter* appears to be the main dominant bacterium, with extremely high relative abundance, reaching 85.80%, 80.85%, 52.57%, and 21.79% in the four samples. In cows with purulent discharge ≥ 50%, *Caviibacter* was not the predominant pathogen, with its relative abundance close to zero. In contrast, genera such as *Fusobacterium* and *Helcococcus* gradually occupied a larger proportion. In cows with foul-smelling watery discharge, *Bacteroides*, *Porphyromonas*, and *Peptostreptococcus* gradually replaced *Fusobacterium* and *Helcococcus* as the dominant genera. Thus, we hypothesize that this may represent a succession of pathogenic bacteria during the development of bovine metritis, resulting from bacterial cooperation, immune dysregulation, and microbial imbalance caused by inflammation [16]. This phenomenon is also commonly observed in diseases like acute diarrhea and periodontitis [17,18].

In China, most dairy farms typically conduct vaginal discharge examinations on postpartum cows on days 5, 10, and 15 after calving, and implement intervention treatments for individuals with discharge scores of 3 or 4. However, in this study, we found that although cows with a score of 2 do not meet the conventional criteria for “severe disease,” the composition and structure of their uterine microbiota have already undergone significant changes compared to healthy individuals with a score of 1, most notably characterized by a high enrichment of *Caviibacter*. According to the latest taxonomic classification, *Caviibacter* belongs to the family Pasteurellaceae and currently includes only one known species—*Caviibacter abscessus*. This bacterium has so far only been isolated from the mandibular lymph nodes and cervical abscesses of guinea pigs [19,20]. Research on *Caviibacter* remains limited, and it was not until recent years that the bacterium was detected in cases of bovine periodontitis and purulent vaginal discharge in dairy cows [21,22]. However, these findings have not attracted much attention and lack systematic investigation. Notably, a previous comparative study of the vaginal and uterine microbiota of Holstein–Friesian and Jersey cows reported the presence of *C. abscessus* in the purulent discharge of Holstein–Friesian cows [21]. Another study reported the detection of *Caviibacter* in Simmental cows, where it was more frequently observed in multiparous individuals [23]. In the present study, we focused on Holstein–Friesian cows and further identified that the high abundance of *Caviibacter* was mainly found in uterine microbiota samples with purulent discharge comprising less than 50%, with a maximum relative abundance reaching 60.25%. This phenomenon suggests that *Caviibacter* may play a key role during the early stages of endometritis, potentially facilitating the subsequent colonization and proliferation of more pathogenic bacteria such as *Fusobacterium* and *Helcococcus*. We speculate that *Caviibacter* may serve as a potential opportunistic pathogen and biomarker in the early phase of purulent endometritis in Holstein cows, offering a theoretical basis for early warning and targeted therapy. To further investigate its pathogenicity, we attempted in vitro isolation and culture. However, due to its strict anaerobic requirements and limitations in laboratory conditions, we have not yet successfully obtained a pure culture of the strain.

Genera such as *Fusobacterium*, *Helcococcus*, *Bacteroides*, *Porphyromonas*, and *Peptostreptococcus* are important members of the microbial communities in the gut, oral cavity, and vagina [24,25,26]. The increased relative abundance of *Fusobacterium*, *Bacteroides*, and *Porphyromonas* has long been considered a hallmark of bovine metritis [8,27,28,29]. Recent studies have shown that the growth of opportunistic pathogens like *Fusobacterium*, *Bacteroides*, and *Porphyromonas* in cows with metritis is related to immune dysregulation and metabolic changes [30]. The observed enrichment of these opportunistic pathogens at different stages may be associated with changes in the uterine microenvironment during the progression of metritis, though further validation is needed. Additionally, increasing reports suggest that *Helcococcus* and *Peptostreptococcus* are also linked to uterine diseases and should not be overlooked [31,32]. Our study supports this view, as we observed higher abundances of *Helcococcus* and *Peptostreptococcus* in cows with purulent discharge greater than 50% and in those with foul-smelling watery discharge. Furthermore, *Helcococcus* has been isolated not only from cows with metritis and mastitis but also from abscesses in the umbilical regions of diabetic patients and intracranial abscesses in patients with cholesteatoma [33,34,35]. Based on our experimental results, we speculate that *Helcococcus* may be more related to abscess formation. *Peptostreptococcus* has been shown to be involved in immune tolerance induction in type I endometrial cancer [36]. We propose that *Peptostreptococcus* may exacerbate uterine inflammation through immune responses, playing a significant role during the most severe stages of metritis. However, further research is needed to explore the pathogenic mechanisms of these characteristic genera in metritis.

In conclusion, we found that the composition of the uterine microbiota and the distribution of specific pathogens in postpartum dairy cows with different vaginal discharge states showed significant differences. These findings provide new insights for the prevention and treatment of metritis. However, this study has certain limitations, such as a limited sample size and the lack of longitudinal data. Although vaginal discharge scoring was used to assess disease severity, uterine health also changes dynamically with days postpartum (DPP. In this study, cows at 15 days postpartum were selected to reflect the common time point for evaluating uterine status in current veterinary clinical practice. However, this time-specific design may limit the generalizability of the findings to other postpartum stages. Future studies will include longitudinal sampling across multiple postpartum time points to better capture the dynamic changes in the uterine microenvironment and disease progression. In addition, microbiota analysis alone is insufficient to fully clarify the pathogenic mechanisms of specific microorganisms. Subsequent research will focus on optimizing the isolation and culture of *Caviibacter* and further investigating its biological characteristics and interactions with the host in order to provide a stronger theoretical basis for understanding its role in the pathogenesis of endometritis and to support more targeted clinical interventions.

## 5. Conclusions

This study demonstrates that the uterine microbiota of postpartum dairy cows significantly changes with different vaginal discharge scores. Cows with purulent or malodorous discharge exhibit lower microbial diversity and increased abundance of potential pathogens such as *Caviibacter*, *Fusobacterium*, *Helcococcus*, *Bacteroides*, *Porphyromonas*, and *Peptostreptococcus*. Notably, *Caviibacter* was enriched in cows with mildly purulent discharge, suggesting its potential role as an early biomarker for uterine infections. These findings enhance our understanding of the microbial dynamics associated with endometritis and may provide new insights for early diagnosis and targeted interventions in dairy cows, contributing to the development of sustainable dairy farming and supporting advancements in modern agricultural animal health management.

## Data Availability

The original contributions presented in this study are included in the article. Further inquiries can be directed to the corresponding authors.
